# An Automated Frailty Index and Loss to Follow-Up Among Patients With Diabetic Retinopathy

**DOI:** 10.1001/jamanetworkopen.2025.6920

**Published:** 2025-04-30

**Authors:** Manuela Gutierrez Velez, Logan Bearfield, Alicia Chen, Margaret Havunjian, Heidi Whiteside, Kathryn Callahan, Joseph Rigdon, Sally S. Ong

**Affiliations:** 1 Department of Ophthalmology, Wake Forest School of Medicine, Winston Salem, North Carolina; 2 Department of Biostatistics and Data Science, Wake Forest Baptist Health, Winston Salem, North Carolina; 3 Department of Internal Medicine, Section on Gerontology and Geriatric Medicine, Wake Forest School of Medicine, Winston Salem, North Carolina

## Abstract

This cohort study examines whether patients with diabetic retinopathy who are frail according to an electronic frailty index have higher rates of loss to follow-up compared with less-frail patients.

## Introduction

Complications from diabetic retinopathy (DR), including diabetic macular edema (DME) and proliferative diabetic retinopathy (PDR), remain the primary causes of vision loss in working-age adults in most developed countries.^1^ In DME, therapeutic outcomes can be limited by inadequate intravitreal anti–vascular endothelial growth factor (VEGF) injection frequency related to loss to follow-up (LTFU).^1^ In PDR, patients LTFU after receiving only anti-VEGF injections have worse outcomes than those receiving panretinal photocoagulation (PRP),^2^ the criterion standard treatment.^3^ Frailty is defined as an aging-related syndrome of physiological decline, marked by vulnerability to adverse health outcomes.^4^ Pajewski et al^4^ tested and validated an electronic frailty index (eFI) at Wake Forest University School of Medicine (WFUSM). In this study, we hypothesize that patients with DR and elevated eFI would have higher rates of LTFU than patients with lower eFI.

## Methods

For this cohort study, patients with DR were identified using *International Classification of Diseases, Ninth Revision *or *International Statistical Classification of Diseases and Related Health Problems, Tenth Revision* codes and were included if they underwent an initial eye examination with a retina specialist at WFUSM between January 1, 2017, and December 31, 2019, and had an available eFI within 2019 to 2021. The WFUSM eFI comprises more than 50 items and is computed on a weekly basis for all patients aged 55 years or older with more than 2 outpatient visits in the past 2 years with recorded blood pressure measurements. Variables included in the eFI are shown in detail in the supplemental tables in Pajewski et al.^4^ The need for informed consent was waived by the WFUSM institutional review board given the use of deidentified data collected retrospectively, in accordance with 45 CFR §46. This study follows the STROBE reporting guidelines.

LTFU was measured through missed visits (ie, failure to return to the clinic for ≥20 days past the recommended follow-up interval).^5^ If there were multiple missed visits, the missed visit after the longest follow-up interval recommended was chosen within 2 years of the initial encounter with ophthalmology. Patients were categorized as fit (eFI <0.1), prefrail (eFI ≥0.1 to ≤0.21), or frail (eFI >0.21). Descriptive statistics were used to characterize patients according to eFI categories. Generalized linear regression models (logistic, linear, and Poisson) were used with and without adjustment for center involving DME on optical coherence tomography, PDR, tractional retinal detachment, age, sex, payer type, and hemoglobin A_1C_ (near baseline). *P* < .05 was considered statistically significant.

## Results

A total of 249 patients (mean [SD] age, 63.5 [8.2] years; 133 female [53.4%]) were included (Table 1). Compared with fit individuals, prefrail individuals had higher odds of LTFU at 3 to 6 months (odds ratio [OR], 4.62; 95% CI, 1.10-19.40; *P* = .04) or more than 6 months (OR, 14.12; 95% CI, 1.75-174.07; *P* = .04); frail individuals had higher odds of LTFU at less than 3 months (OR, 3.76; 95% CI, 1.33-10.64; *P* = .01), 3 to 6 months (OR, 8.44; 95% CI, 1.63-43.69; *P* = .01), or more than 6 months (OR, 23.35; 95% CI, 1.41-385.66; *P* = .03) (Table 2). Compared with fit individuals, prefrail (incidence rate ratio, 0.45; 95% CI, 0.36-0.58; *P* < .001) and frail (incidence rate ratio, 0.15; 95% CI, 0.12-0.20; *P* < .001) individuals had significantly lower rates of anti-VEGF injections. Compared with fit individuals, frail individuals had higher odds of LTFU due to death (OR, 7.59; 95% CI, 2.39-30.56; *P* = .002).

**Table 1.** Patient Demographics, Systemic and Ocular Characteristics (for the Worse Affected Eye), by eFI Category

Characteristic	Patients, No. (%)			*P* value^a^
	Fit (eFI <0.1) (n = 65)	Prefrail (eFI ≥0.1 to ≤0.21) (n = 108)	Frail (eFI >0.21) (n = 76)	
Age, mean (SD), y	62.7 (8.1)	63.4 (7.7)	64.5 (8.9)	.42
Sex				
Male	36 (55.4)	53 (49.1)	27 (35.5)	.05
Female	29 (44.6)	55 (50.9)	49 (64.5)	
Race^b^				
Asian	2 (3.1)	3 (2.8)	2 (2.6)	.99
Black	16 (24.6)	28 (25.9)	22 (28.9)	
White	44 (67.7)	73 (67.6)	50 (65.8)	
Other^c^	3 (4.6)	4 (3.7)	2 (2.6)	
Ethnicity^b^				
Hispanic	4 (6.2)	5 (4.6)	2 (2.7)	.60
Non-Hispanic	61 (93.8)	103 (95.4)	73 (97.3)	
Language				
English	61 (93.8)	101 (93.5)	72 (96.0)	.98
Spanish	3 (4.6)	4 (3.7)	2 (2.7)	
Other^d^	1 (1.5)	3 (2.8)	2 (2.6)	
Payer type				
Commercial	4 (6.2)	3 (2.8)	0	
Managed care	32 (49.2)	45 (41.7)	20 (26.3)	<.001^e^
Medicaid	1 (1.5)	9 (8.3)	7 (9.2)	
Medicare	20 (3.8)	47 (43.5)	47 (61.8)	
Other government programs	8 (12.3)	4 (3.7)	2 (2.6)	
Diabetes type				
1	3 (4.6)	13 (12.0)	6 (7.9)	.26
2	62 (95.4)	95 (88.0)	70 (92.1)	
Hemoglobin A_1C_, mean (SD), %	8.9 (2.5)	7.9 (2.0)	8.2 (2.0)	.03^e^
Insulin use				
Yes	44 (67.7)	75 (69.4)	59 (77.6)	.36
No	21 (32.3)	33 (3.6)	17 (22.4)	
Worse affected eye				
Right eye	24 (36.9)	52 (48.1)	36 (47.4)	.32
Left eye	41 (63.1)	56 (51.9)	40 (52.6)	
Visual acuity, mean (SD), logMAR	0.62 (0.61)	0.61 (0.67)	0.64 (0.62)	.95
Intraocular pressure, mean (SD), mm Hg	15.9 (4.5)	15.3 (3.5)	16.6 (3.8)	.10
Lens status				
Phakic	44 (67.7)	66 (61.1)	38 (5.0)	.10
Pseudophakic	21 (32.3)	42 (38.9)	38 (5.0)	
Type of DR				
Mild NPDR	12 (18.5)	19 (17.6)	15 (19.7)	.93
Moderate NPDR	17 (26.2)	33 (3.6)	21 (27.6)	
Severe NPDR	5 (7.7)	8 (7.4)	9 (11.8)	
Proliferative DR	31 (47.7)	48 (44.4)	31 (4.8)	
Presence of diabetic macular edema				
Yes	42 (64.6)	63 (58.3)	44 (57.9)	.68
No	23 (35.4)	45 (41.7)	32 (42.1)	
Presence of vitreous hemorrhage, preretinal hemorrhage, or large neovascularization of the disc (>1/4 disc diameter in size)				
Yes	19 (29.2)	28 (25.9)	16 (21.1)	.54
No	46 (7.8)	80 (74.1)	60 (78.9)	
Presence of neovascularization of the iris or neovascular glaucoma				
Yes	5 (7.7)	1 (0.9)	3 (3.9)	.06
No	60 (92.3)	107 (99.1)	73 (96.1)	
Presence of macula or fovea involving tractional retinal detachment				
Yes	9 (13.8)	9 (8.3)	3 (3.9)	.12
No	56 (86.2)	99 (91.7)	73 (96.1)	
Presence of other ocular comorbidities				
Yes	15 (23.1)	36 (33.3)	28 (36.8)	.18
No	50 (76.9)	72 (66.7)	48 (63.2)	

Abbreviations: DR, diabetic retinopathy; eFI, electronic frailty index; NPDR, nonproliferative diabetic retinopathy.

SI conversion factor: To convert hemoglobin A_1C_ to proportion of total hemoglobin, multiply by 0.01.

^a^ *P* values were calculated with 1-way analysis of variance for continuous variables (eg, age) and with Fisher exact test for categorical variables (eg, language).

^b^ Patient race and ethnicity were self-reported and were obtained from the electronic health record. Race and ethnicity were assessed to determine whether there were differences across the 3 eFI groups.

^c^ Other race includes American Indian or Alaska Native, Native Hawaiian or Other Pacific Islander, other, unknown, choose not to disclose, unable to obtain, or 2 or more races.

^d^ Other language includes Vietnamese, Urdu, or other languages.

^e^ Denotes statistically significant *P* value.

**Table 2.** Estimates of Associations Between eFI Group (Fit, Prefrail, and Frail) and Outcomes of Interest^a^

Variable	Unadjusted estimate (95% CI)	*P* value	Adjusted estimate (95% CI)^b^	*P* value
Missed visits after a recommended follow-up interval (reference: none)				
Prefrail vs fit				
<3 mo^c^	1.96 (0.92 to 4.15)	.08	2.26 (0.95 to 5.35)	.06
3-6 mo^c^	2.78 (0.89 to 8.69)	.08	4.62 (1.10 to 19.40)	.04^c^
>6 mo^c^	8.89 (1.01 to 78.18)	.05	14.12 (1.75 to 174.07)	.04^c^
Frail vs fit				
<3 mo^c^	3.04 (1.25 to 7.41)	.01^c^	3.76 (1.33 to 10.64)	.01^c^
3-6 mo^c^	3.70 (1.03 to 13.35)	.05	8.44 (1.63 to 43.69)	.01^d^
>6 mo^c^	11.11 (1.13 to 109.35)	.04^d^	23.35 (1.41 to 385.66)	.03^d^
Total follow-up duration, mo (reference: fit)				
Prefrail^e^	−1.60 (−4.68 to 1.48)	.31	−1.18 (−4.54 to 2.18)	.49
Frail^e^	−1.45 (−4.77 to 1.86)	.39	−0.84 (−4.53 to 2.86)	.65
LogMAR at 1 y (reference: fit)				
Prefrail^e^	0.04 (−0.24 to 0.32)	.78	0.09 (−0.22 to 0.39)	.57
Frail^e^	0.14 (−0.16 to 0.44)	.36	0.28 (−0.04 to 0.61)	.09
LogMAR at 2 y (reference: fit)				
Prefrail^e^	−0.11 (−0.40 to 0.18)	.47	−0.10 (−0.39 to 0.18)	.46
Frail^e^	0.07 (−0.23 to 0.37)	.65	0.13 (−0.18 to 0.44)	.40
DR worsened (reference: fit)				
Prefrail^c^	0.63 (0.31 to 1.31)	.21	0.79 (0.33 to 1.87)	.58
Frail^c^	0.69 (0.30 to 1.55)	.37	0.81 (0.31 to 2.14)	.67
Anti-VEGF injections (reference: fit)				
Prefrail^f^	0.43 (0.36 to 0.51)	<.001^d^	0.45 (0.36 to 0.58)	<.001^d^
Frail^f^	0.45 (0.37 to 0.55)	<.001^d^	0.15 (0.12 to 0.20)	<.001^d^
Return to clinic after LTFU (reference: fit)				
Prefrail^c^	0.58 (0.28 to 1.20)	.14	0.79 (0.33 to 1.87)	.58
Frail^c^	0.63 (0.29 to 1.34)	.23	0.81 (0.31 to 2.14)	.67
Hospitalization during LTFU (reference: fit)				
Prefrail^c^	1.28 (0.40 to 4.90)	.69	1.29 (0.35 to 5.51)	.17
Frail^c^	2.96 (0.99 to 10.98)	.07	2.58 (0.70 to 11.40)	.18
LTFU due to death (reference: fit)				
Prefrail^c^	3.05 (0.98 to 9.45)	.05	2.88 (0.92 to 11.21)	.09
Frail^c^	7.04 (2.29 to 21.60)	<.001^d^	7.59 (2.39 to 30.56)	.002^d^

Abbreviations: DR, diabetic retinopathy; eFI, electronic frailty index; LTFU, loss to follow-up; VEGF, vascular endothelial growth factor.

^a^ We used multinomial logistic regression for comparison of odds of missed visit time category, linear regression for comparing duration of follow-up (months) and visual acuity outcomes (logMAR at 1 and 2 years), logistic regression for worsening DR, return to clinic, hospitalization, and death and proportional odds logistic regression for the total number of anti-VEGF injections among eFI categories.

^b^ Adjusted for presence of center involving diabetic macular edema, proliferative DR, tractional retinal detachment, age, sex, payer type, and hemoglobin A_1C_ near baseline.

^c^ Denotes odds ratio.

^d^ Denotes statistically significant *P* value.

^e^ Denotes β coefficient.

^f^ Denotes incidence rate ratio.

## Discussion

This cohort study highlights that frail and prefrail patients with DR have higher rates of LTFU and lower rates of anti-VEGF injections than fit patients. Furthermore, frail patients have higher odds of LTFU due to death. Visual outcomes in DME have been correlated with anti-VEGF treatment intensity.^1^ Frail and prefrail patients may especially benefit from sustained delivery of anti-VEGF via novel treatment options that emerge in the future. For frail and prefrail patients with PDR and a high risk of LTFU, it may be advantageous to favor PRP over anti-VEGF injections, and when these patients have vitreous hemorrhage, early vitrectomy with PRP could be considered.^2,6^ This study is limited by its retrospective design and the risk of selection bias. Large prospective studies that further examine reasons for LTFU are needed in the future to validate the clinical utility of these results. In summary, this study suggests that the eFI can be used to estimate LTFU in patients with DR.

## Notes

Supplement.
Data Sharing Statement
