# Supplementary material for: An Automated Frailty Index and Loss to Follow-Up Among Patients With Diabetic Retinopathy
Source: JAMA Netw Open. 2025 Apr 30;8(4):e256920. doi: 10.1001/jamanetworkopen.2025.6920 (PMC12044481; doi:10.1001/jamanetworkopen.2025.6920)
Supplement: Supplement. — Data Sharing Statement [file jamanetwopen-e256920-s001.pdf]

Data Sharing Statement

Gutierrez Velez. An Automated Frailty Index and Loss to Follow-Up Among Patients With Diabetic Retinopathy. *JAMA Netw Open*. Published April 30, 2025.
doi:10.1001/jamanetworkopen.2025.6920

Data
Data available: Yes
Data types: Deidentified participant data, Data dictionary
How to access data: song@wakehealth.edu
When available: With publication

Supporting Documents
Document types: Statistical/analytic code
How to access documents: song@wakehealth.edu
When available: With publication

Additional Information
Who can access the data: Researchers whose proposed use of the data has been approved
Types of analyses: For a specified purpose
Mechanisms of data availability: After approval of a proposal and with a signed data access agreement
